# Cerebellar stroke complicating coronary catheterization: a case report

**DOI:** 10.11604/pamj.2021.40.172.32031

**Published:** 2021-11-19

**Authors:** Hamza Chraibi, Zakia El Yousfi, Najat Mouine, Zouhair Lakhal, Aatif Benyass

**Affiliations:** 1 Cardiology Department, Mohammed V Military Instruction Hospital, Mohammed V University, Rabat, Morocco,; 2 Radiology Department, Mohammed V Military Instruction Hospital, Mohammed V University, Rabat, Morocco

**Keywords:** Coronary angiography, percutaneous coronary intervention, ischemic stroke, case report

## Abstract

Cerebrovascular events are rare but devastating events that can complicate any coronary intervention. In the vast majority of cases, they involve major cerebral arteries. We report the case of a 56-year-old woman admitted for unstable angina associated with severe left systolic dysfunction. She developed moderate cerebellar stroke while undergoing percutaneous coronary intervention, with a national institutes of health stroke scale score of 5. Immediate systemic thrombolysis was performed, but her neurological status deteriorated. A large hemorrhagic transformation was then diagnosed, and she died despite surgical intervention. Periprocedural strokes are marred with high morbidity and mortality, therefore preventionis key, as many risk factors can be controlled or mitigated. Our patient presented many of these factors; they can be procedure-related (transfemoral approach, anticoagulation) or patient-related (age, diabetes mellitus, uncontrolled hypertension, diffuse atherosclerosis).

## Introduction

Coronary angiography (CA) and percutaneous coronary intervention (PCI) have become irreplaceable diagnostic and therapeutic tools in the context of coronary artery disease (CAD); yet they are not without risks. Cerebrovascular events are rare but serious accidents that can complicate any intervention, as the use of mechanical devices (catheters and wires) may dislodge atherosclerotic debris from the aorta, leading to cerebral embolization and ischemia. Coronary catheterization-related ischemic stroke (CC-IS), although its incidence is low according to recent studies (0.05 - 0.1% after CA [1] and 0.2 to 0.3% after PCI [2-5]), is associated with a high mortality rate (20 to 40%) and decreased quality of life [1-4,6]. Management of this complication is especially delicate, due to the associated cardiopathy and the numerous comorbidities these patients usually share, such as diabetes mellitus and hypertension.

In about 90% of patients, CC-IS involves major arterial territories with a cerebral distribution. In this paper, we present a rare case of a woman who developed cerebellar CC-IS following non-emergent coronary catheterization.

## Patient and observation

**Patient information:** a 56-year-old woman, whose history included diabetes mellitus, uncontrolled hypertension, and dyslipidemia, complained of chest pain beginning 10 years ago, with poor medication adherence. Three months before, symptoms aggravated as both chest pain intensity and duration increased, along with new-onset shortness of breath on exertion. It was decided to admit her in the cardiology department for further exploration.

**Clinical findings:** initial physical examination showed stable vital signs along with grade II hypertension. Auscultation found no rales or murmur. There was no peripheral edema or other signs of heart failure. Abdominal examination revealed no tenderness, hepatomegaly, or ascites. Electrocardiogram at admission showed sinus rhythm with complete left bundle branch block. Chest radiograph found an enlargement of the cardiac silhouette.

**Diagnostic assessment:** biological results included moderate renal failure with a creatinine level of 12 mg/L. Hematology was normal (hemoglobin: 13.1 g/dL, white blood cells: 6,300/µL and platelets: 273,000/µL). Metabolic workup showed good diabetic control (HbA1c: 5.7%) and dyslipidemia (LDLc: 1.28 g/L, triglycerides: 2.18 g/L). All drawn troponins were negative; thus unstable angina was the most probable diagnosis. A transthoracic echocardiographic was performed, demonstrating a dilated left ventricle, with anterior wall motion abnormalities and severe systolic dysfunction (ejection fraction = 31%). Echo-Doppler examination of the supra-aortic trunks showed diffuse atheromatous lesions in the carotid arteries, without significant stenoses. We decided to uptitrate her medication to reach guideline-directed optimal therapy and she was scheduled for coronary angiography. Her treatment included at the time aspirin 75 mg o.d., ramipril 5 mg o.d., spironolactone 50 mg o.d., bisoprolol 10 mg o.d., furosemide 40 mg o.d., atorvastatin 40 mg o.d., trimetazidine 35 mg b.i.d. and insulin.

**Figure 1:**
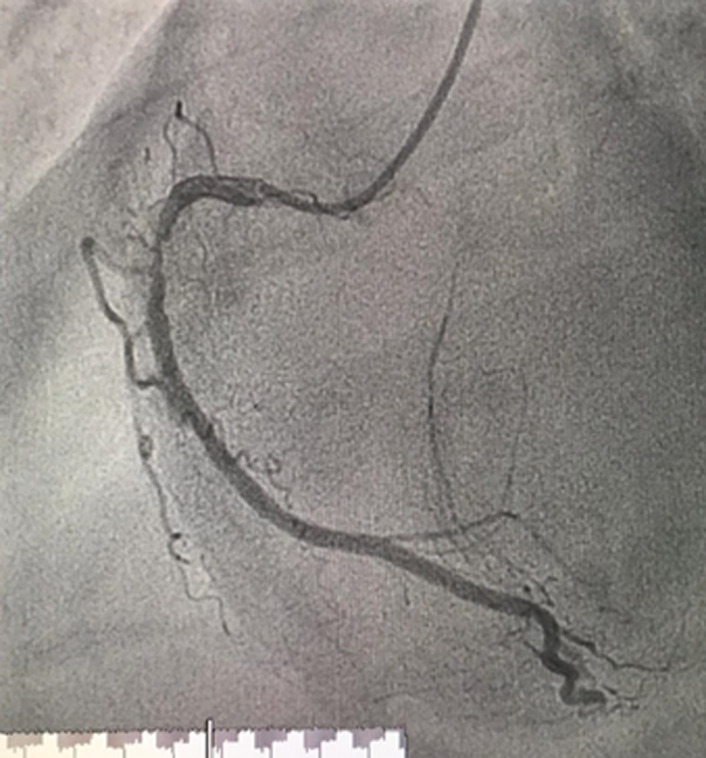
coronary angiography showing a significant lesion of the mid right coronary artery

**Figure 2:**
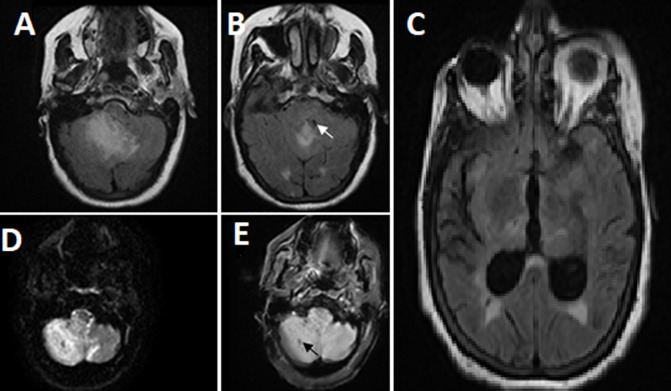
brain MRI revealing a right cerebellar stroke with evidence of hemorrhagic infarction: (A,B,C) FLAIR axial slice demonstrates a hyperintense right cerebellar cortico-subcortical signal with a discrete mass effect on the fourth ventricle, with moderate tri-ventricular hydrocephalus (arrow); D) diffusion imaging in axial section shows a hyperintense signal in the infarcted region in relation to a decrease in apparent diffusion coefficient; E) T2 gradient echo shows a small hypointense focus in the right cerebellum indicating a small hemorrhagic transformation (arrow)

Three days later, the patient underwent coronary angiography, with transfemoral access. Results showed a significant lesion of the mid right coronary artery (Figure 1). Before angioplasty could be performed, the patient complained of severe headaches and vertigo. She then developed sudden, brief clonic contractions of the upper extremities. The procedure was interrupted, and neurology was consulted. The patient was transferred to the intensive care unit. Examination found an altered neurological status with confusion and dysphasia (but no motor deficit or visual impairment), along with severe hypertension (210/110 mmHg). Her National Institutes of Health Stroke Scale (NIHSS) score was 5, indicating a moderate stroke. A brain magnetic resonance imaging (MRI) revealed a right cerebellar stroke with evidence of hemorrhagic infarction (Figure 2).

**Therapeutic interventions:** immediate systemic thrombolysis with tenecteplase was performed, three hours after symptom onset, with no significant improvement.

**Follow-up and outcome of interventions:** on the day following the incident, a deteriorating level of consciousness prompted a brain computed tomography which diagnosed a large hemorrhagic transformation. A craniotomy was performed, but the patient died two days later.

**Informed consent:** it was obtained from the patient´s next of kin (her son).

## Discussion

As stated previously, CC-IS is a serious complication marred by high morbidity and fatal in about a third of cases. It represents the third most common cause of death in patients undergoing PCI and accounts for 4.1% of all in-hospital PCI-related deaths [7]. Potential risk factors include advanced age, diabetes mellitus, hypertension, left ventricular systolic dysfunction, history of stroke, chronic kidney disease, and emergent procedure. Procedure length, transfemoral access, and inadequate anticoagulation may also play a role [2-6]. Our patient presented many of those factors. She also had advanced atherosclerosis, making for a riskier-than-average procedure.

Our patient´s uncontrolled arterial hypertension may have played a major role. On the morning of a procedure, white coat effect, urinary retention, pain and anxiety increase blood pressure and exacerbate this phenomenon. Hypertension also distorts blood vessels, causing technical failure and major procedural complications [8]. Cerebellar CC-IS is highly unusual, and to our knowledge, has only been reported in a select few cases [9,10]. Fuchs *et al*. in their series of forty-one patients, observed that the culprit artery was the middle cerebral artery in 47.6%, the posterior cerebral artery in 23.8% and the anterior cerebral artery in 9.6% of cases [4]. In Dukkipati *et al*. the distribution was similar (middle: 56% of patients, posterior: 37%, anterior: 2%). The basilar and cerebellar arteries were involved in 7%, 5%, and 5% of cases respectively. Only 7% of patients had infarcts involving more than one artery distribution [2].

Vascular anatomy helps us understand the rarity of this phenomenon. The embolus originating from the aortic arch must take a long and difficult path to the cerebellar arteries, passing through the subclavian artery for left-sided lesions or brachiocephalic trunk for right-sided lesions, then the vertebral and basilar arteries in either case. Therefore, in about 90% of cases, CC-IS involves one of the major cerebral arteries instead. Sometimes, when transfemoral CC is performed, the catheter enters the subclavian artery. It is likely that this patient had a plaque at the orifice of the left vertebral artery that was dislodged by the catheter, sending embolic material occluding the basilar artery. Because CC-IS is so rare, management is not clearly codified. The 2018 American guidelines recommend thrombolysis for all acute stroke patients within 3 hours of last known normal [11]. Tissue plasminogen activator (tPA) is the thrombolytic drug of choice. Intra-arterial injection is the most reported method, with good outcomes across the literature [12]. However, when the patient´s critical condition amends immediate treatment, systemic injection should be considered for its immediate availability, providing hemorrhage has been ruled out by imaging. Serry *et al*. have reported such a case where the patient fully recovered following systemic thrombolysis [13].

When administering thrombolytic therapy, it is wise to take into consideration the hemorrhagic risk, especially in patients undergoing CC, as they already received anticoagulant therapy before or during the intervention. The risk of hemorrhagic transformation has never been studied in large studies, but small series have reported a rate similar to non procedure-related strokes (10 to 15%) [14]. Access-site bleeding is also a concern, especially in transfemoral interventions, but the interventional management of stroke study has shown that these incidents are quite rare (3.3%) and can be prevented using local compression devices [15]. Thus, thrombolysis has been deemed mostly safe in the context of CC-IS.

Cerebral imaging is mandatory before reperfusion to exclude hemorrhage, but many voices have raised concern over the management delay this can cause. De Marco *et al*. have proposed immediate angiography as the diagnostic and therapeutic method of choice, as it led to better outcomes in their series of eight patients [16]. The technique also allows for the administration of local thrombolytic therapy in select cases. Chan *et al*. also vouched for immediate interventional treatment, consisting of catheter-based intra-arterial thrombolysis with balloon angioplasty [17]. An experienced cerebral angiographer is needed, and this method warrants larger-scale studies before being applied in common practice.

Prognosis of CC-IS is poor. Mortality rates range from 22 to 37% in recent registries. Quality of life is severely reduced, as 72% of patients have persisting neurological deficits and will need skilled homecare or assisted living [2]. The economic impact of CC-IS is also tremendous, by extending the patient´s postprocedural hospital stay by several days or weeks [4]. Therefore, prevention is key; extra precautions should be taken with high-risk patients, or when the procedure is done emergently. Anticoagulation should be optimally dosed and transradial access should be favored [5].

## Conclusion

CC-IS is an uncommon but devastating event associated with substantial morbidity and mortality. Cerebellar infarction is an uncommon form in this context. Optimal management remains controversial, underlining the urgent need for evidence-based guidelines. Prevention is of the utmost importance; procedure-wise, transradial approach should be favored, with adequately sized material and careful manipulation of catheters and wires. Patients should be on optimal medical therapy before the intervention to reduce arterial hypertension and thromboembolic risk. Efforts should also be made to reduce anxiety, by comforting patients or sedating them in extreme cases.
